# Phenol and chromone compounds for *in silico* inhibition of nsP2 and nsP3 of Chikungunya virus

**DOI:** 10.1016/j.pscia.2025.100084

**Published:** 2025-07-25

**Authors:** Joan Petrus Oliveira Lima, Caio Henrique Alexandre Roberto, Matheus Nunes da Rocha, Victor Moreira de Oliveira, Rafael Melo Freire, Ralph Santos-Oliveira, Emmanuel Silva Marinho, Pedro de Lima Neto, Pierre Basílio Almeida Fechine

**Affiliations:** a Advanced Materials Chemistry Group (GQMat), Department of Analytical Chemistry and Physical Chemistry, Federal University of Ceará – UFC, Campus do Pici, CP 12100, 60451-970, Fortaleza, CE, Brazil; b Programa de Pós-Graduação em Ciências Naturais, Universidade Estadual do Ceará – UECE, Centro de Ciências e Tecnologias – CCT, Av. Dr. Silas Munguba, 1700 – Campus do Itaperi, Fortaleza, CE, CEP 60.714-903, Brazil; c Universidad Central de Chile, 8330601, Santiago, Chile; d Brazilian Nuclear Energy Commission, Nuclear Engineering Institute, Laboratory of Nanoradiopharmaceuticals and Synthesis of Novel Radiopharmaceuticals, Rio de Janeiro, 21941906, Brazil

**Keywords:** Molecular docking, Neglected tropical disease, Chikungunya virus, Natural products, *Daldinia*

## Abstract

The rising concern about neglected tropical diseases imposes a global challenge, in this sense, this work brings 12 potential candidates based on chromone and phenol compounds to inhibit nsP2 and nsP3 of the Chikungunya virus (CHIKV), through molecular docking and ADMET evaluation. The molecular docking simulations for the nsP2 showed mild binding, in the nsP3 all the derivatives presented −6 ​kcal/mol binding affinity and interacts with crucial residues in the replication cycle of CHIKV, the 5 best were chosen as the main derivatives for absorption, distribution, metabolism, excretion and toxicity (ADMET) evaluation). The ADMET results show high drug-likeness values, with good oral and intestinal absorption, excretion, distribution and toxicity, with moderate (Der9 to Der12) and poor (Der8) metabolism. Therefore, the 5 derivatives are potential candidates to treat chikungunya.

**Table:** 

Abbreviation	Definition
ADMET	Absorption, Distribution, Metabolism, Excretion, Toxicity
CHIKV	Chikungunya Virus
FDA	Food and Drug Administration
MCE-18	Medicinal Chemistry Evolution in 2018
nsP2	Non-Structural Protein 2
nsP3	Non-Structural Protein 3
NTD	Neglected Tropical Disease
PDB	Protein Data Bank
QED	Quantitative Estimated Drug-likeness
RMSD	Root Mean Square Deviation
XRD	X-Ray Diffraction
ZIKV	Zika Virus

## 1 Introduction

Neglected tropical diseases (NTDs) are an ever-increasing world concern due to the impact in tropical and subtropical countries that struggles to eradicate them, but mostly due to climate change that may spread NTDs to novel regions and the possibility of new epidemics and pandemics. Among the NTDs, Chikungunya fever, a disease caused by the namesake virus (CHIKV), an arbovirus like dengue and zika, which is transmitted mainly by *Aedes Aegypti* and *Aedes Albopticus*, common in tropical weather regions, especially during rainy season and close to wildlife that participate in the mosquitoes cycle [[1], [2], [3]].

Dengue, zika and chikungunya have fever and rash as common symptoms, but dengue and chikungunya usually causes arthritis, arthralgia and joint pain [4,5], but differs in the higher lethality of dengue [6] and the frequent chronic pain caused by chikungunya [7]. Due to the lack of antiviral for these diseases, the traditional public health actions are to prevent one or more steps of the cycle of *Aedes* mosquitoes (entomological surveillance and vector control) and treat the symptoms of the infected. In Brazil, despite its efficiency [8], the epidemic persists, attributed to the role of the population in the vector control and the intersectoral of the health system to eradicate and treat the diseases [9].

Proposals for treatment and vector control have arisen, the majority are the genetic modification of *Aedes* [10,11], development of vaccines, one recently approved by FDA [12], and antivirals. In the sense of the latter, the *in vitro* testing with already known antiviral compounds, as interferon alpha, ribavirin, favipiravir, chloroquine, arbidol and amantadine, among others repurposed drugs showed promising results. Nonetheless, some are not approved yet to use in humans, as their concentrations are too high for an effective treatment [13,14] or with some restrictions on the administration and effects [15]. A viable approach is the development of novel CHIKV specific drugs through computational drug design, targeting a protein that plays a crucial role in the replication cycle of CHIKV, that may find a potential candidate to inhibit quickly, at the expense of starting the *in vitro* and *in vivo*, in contrast to repurposed drugs [16]. From the possible targets, the nsP2 and nsP3 (non-structural proteins) enzymes perform important roles in the viral replication ok CHIKV, as the nsP2 exhibits phosphatase helicase and proteolytic activities vastly reported on literature, and the nsP3, although not completely elucidated, it's critical for replication and assembly [16,17]. Therefore, the inhibition of those enzymes may be a possible treatment for Chikungunya infection.

Natural compounds usually shows less side effects, are cheaper and easy to obtain when compared to synthetic ones [18], in the CHIKV context, the works of Puranik et al. [19] and Indu et al. [20] bring the computational docking with natural compounds, the first targets the nsP3 with flavonoid derivatives, the second targets the envelope glycoprotein and nsP2 with phytocompounds from online databases, both works showed high potential for inhibition or as a molecular scaffold to novel drugs. So, it's reasonable to commence from natural compounds that showed antiviral activity *in vitro* against similar targets or viruses.

Therefore, our proposal is to evaluate *in silico* the antiviral activity in the nsP2 and nsP3 macrodomains of CHIKV and the absorption, distribution, metabolism, excretion and toxicity (ADMET) of the chromone and phenol compounds extracted from *Daldinia* sp., molecules from the work of Zhang and coworkers [21], as these natural compounds presented anti-ZIKV and anti-influenza A virus activity, some are relatively small molecules that may interact with the targets, as described in the work of Zhang et al. [22], that was able to inhibit the nsP3. Also, the possibility to achieve a compound that is able to inhibit both CHIKV and ZIKV, which are similar diseases from the same vector, is highly desirable.

## 2 Methodology

### 2.1 Preparation of proteins

**Fig. 1:**
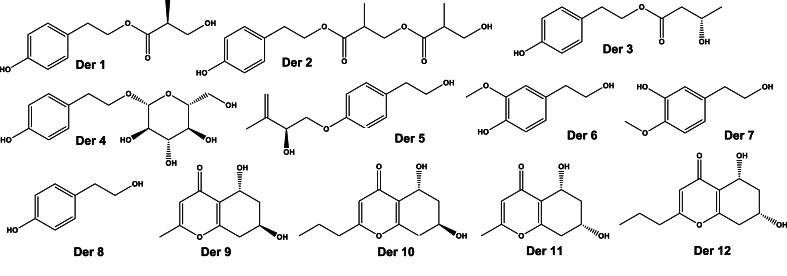
Chromone and phenol derivatives molecules.

The chromone and phenols molecules for Docking and ADMET, Fig. 1, is based on the work of Zhang et al. [21], which extracted and characterized the compounds from *Daldinia* sp. and tested their antiviral properties against zika virus (ZIKV) and influenza A virus. In this sense a computational simulation may indicate a potential anti-CHIKV for future *in vitro* testing.

Those molecules can be classified as phenolic (Der1 to Der8) and chromone (Der9 to Der12) based compounds. It's worth pointing that just the Der1, Der2 Der3, Der9 and Der10 are novel molecules, based on PubChem database, but some compounds, Der4 and Der8, are extensively reported. Der8 is tyrosol, a metabolite from plants and fungi, which is of medical interest due to antioxidant, cardio and neuroprotective properties [23]. Der4 is the glycoside salidroside, found on golden root, that also has neuroprotective activity and nerve regeneration [24] and potential to interrupt the viral cycle of dengue [25].

### 2.2 Docking

Molecular docking simulations were performed to evaluate the potential action of the novel derivatives of phenol and chromones molecules in the replication enzymes nsP2 and nsP3 of CHIKV. The structures were obtained from the repository Protein Data Bank (PDB) and identified as “Structure of Chikungunya virus nsP2 protease” (PDB 3TRK), submitted without mutations with 2.4 ​Å of resolution determined by x-ray diffraction (XRD), classified as a hydrolase expressed in *Escherichia coli* BL21 [26] and “Crystal structure of macro domain of Chikungunya virus” (PDB 3GPG) with 1.65 ​Å resolution determined by XRD, expressed in *Eschericia coli* without mutations [27].

The AutoDock Vina software was used to perform the molecular docking simulations, set to execute a Lamarckian Genetic Algorithm [28]. The grid box for nsP2 was centered in 16.472, 24.972 and 24.389 ​Å for the x, y and z axis respectively with size parameters of 116 ​Å (x), 126 ​Å ​(y) and 120 ​Å (z), the nsP3 in the coordinates 16.667 (x), −25.139 (y) and −1.722 (z) Å with size parameters of 126 ​Å (x), 126 ​Å ​(y) and 120 ​Å (z). To further enhance the pocket, the grids were confirmed with DoGSiteScorer [[29], [30], [31]], to determine the potential active sites based on the protein structure. The DoGSiteScorer was used to evaluate only the pockets, without ligands, with properties and druggability on all the chains (A, B, C and D, given by the PDB). To select the pockets, a simple score of 0.4 or above was used.

As criteria for the protein structure, the methodology proposed by Yan and coworkers [32], which were added Gasteiger charges and essential hydrogen atoms, then removed water molecules. The code for the preparation was made through AutoDockTools [33]. A total of 30 independent simulations were realized, being able to obtain 20 poses per simulation.

To improve the partial refinement of each calculus, the exhaustiveness criteria was set to 64, all bonds and torsion of the ligands were adjusted to twist while the protein structure was kept rigid [34]. As selection criteria for the best pose, the root mean square deviation (RMSD) was carried, with ideal valor bellow 2 ​Å [35]. To evaluate the stability of the complex binder-ligand through the simulations, the energy affinity (ΔG) was used, with desirable values close to −6.0 ​kcal/mol [36].

### 2.3 ADMET

For the ADMET evaluation, an approach similar to the work of Rocha [37], Lima [38] and Lima [39] was used, as the online websites utilizes different drug databases and algorithms, a consensus based on the numerical (like physico-chemical descriptors), empirical (*in vitro* assays) and literature (publications of the already known molecules) can be made to predict the properties of the drugs.

The compounds were drawn in MarvinSketch v22.22 as described in the work of Zhang and coworkers [21], then converted to a SMILES format and.mol to submit to ADMETlab 2.0 (https://admetmesh.scbdd.com/) ADMETboost (https://ai-druglab.smu.edu/admet) and SwissADME (http://www.swissadme.ch/). In order to produce a.mol file in MarvinSketch, the force field used was the MMFF94, with maximum number of conformers of 10.

For a more accurate result, AI Drug Lab (https://ai-druglab.smu.edu/) was used to further improve the results of the consensus, due to the recent implementation of the ADMETboost algorithm trained in the Therapeutics Data Commons ADMET Benchmark, achieving high rank in several tasks and also has a higher degree of specificity in the results [40], and also, uses the same limits for optimal data as ADMETlab 2.0, then being easily comparable.

For druglikeness, the Quantitative Estimation of Druglikeness (QED) and Medicinal Chemistry Evolution 2018 (MCE-18) were performed with the rules of Pfizer (logP <3 and 40 ​< ​TPSA <90 ​Å^2^) [41], GSK (MW ​≤ ​400 ​g/mol, logP ≤4) [42] and the Golden Triangle (GT) rule (200 < MW ​≤ ​500 ​g/mol, −2 ​< ​logD ≤5) [43], from the ADMETlab 2.0.

The metabolism sites, reactive fragments and excretion descriptors were determined from XenoSite (https://xenosite.org/) and ADMETlab 2.0 webservers, while the toxicity were evaluated at pkCSM (https://biosig.lab.uq.edu.au/pkcsm/) and ProTox II (https://tox-new.charite.de/protox_II/).

## 3 Results and discussion

### 3.1 Molecular docking

**Fig. 2:**
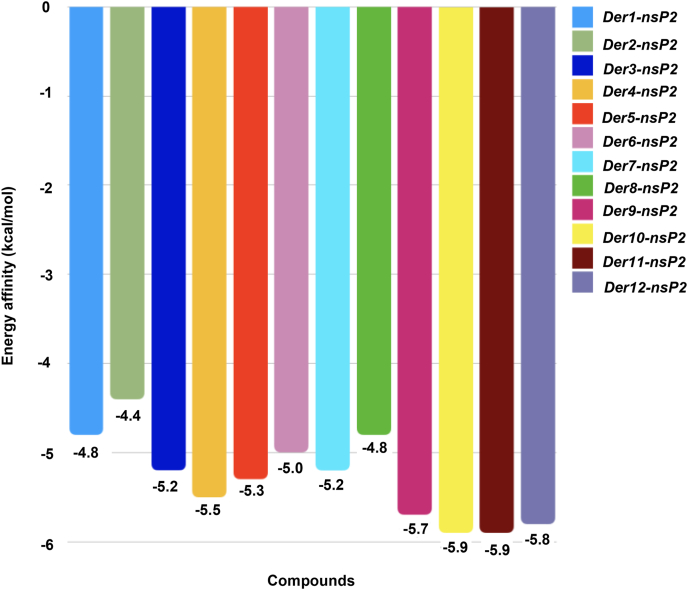
Energy affinity for the complex nsP2-ligands (Der1 to Der12).

Fig. 2 presents the energy affinity of the compounds with the nsP2. The majority of the complexes showed weak interactions (above −6.0 ​kcal/mol), indicating the best compounds being Der9 to Der12, as they have similar structure, in which the stereoisomerism in the carbon 7 (R or S) and the length of the alkyl in the carbon 2 (methyl or propyl) differs. The weakest interaction of these four is the Der9 (−5.7 ​kcal/mol), which has a methyl and R carbon, in contrast, Der11 (−5.9 ​kcal/mol) has a S carbon, indicating that the receptor may be sensitive to stereoisomers. Der12 (−5.8 ​kcal/mol) and Der11 (−5.9 ​kcal/mol) presents the same stereoisomerism, but the former has a propyl in carbon 2 and the latter a methyl, the small difference may indicate a small pocket in the receptor site or to achieve the lowest energy affinity a long molecule may be preferable.

It's important to note that the nsP2 is a well-studied target for molecular docking of CHIKV, in the works of Bassetto et al. [44] and Das et al. [45] long compounds were obtained with rich π-stacking sites with potential inhibitory of nsP2, however in the *in vitro* assays the first compound of Bassetto was unable to inhibit, but some of their derivatives and the best compound of Das were able to, which besides the importance of biological assays to reinforce computational simulations, small molecules with few π-stacking sites may not be suited for the inhibition of nsP2, a possible explanation for the performance of the compounds presented. Similarly, in the work of Indu et al. [20], natural compounds (astragalosides II, III and IV) presented binding energies below −9.5 ​kcal/mol, although the compounds are large and lack sites for π-stacking, they are rich in hydroxyl groups that produce HB interactions with the residues (Tyr1079, Lys1045, Gly1176, His1222, Lys1239). Then, long and rich HB sites molecules have a higher probability to inhibit the nsP2 than small molecules (even if its rich in HB sites), reason that explains the behavior of Der4, and the influence in the binding energy *in silico* is higher, in decrescent order, for the size of the molecule, number of HB sites and π-stacking sites.

**Fig. 3:**
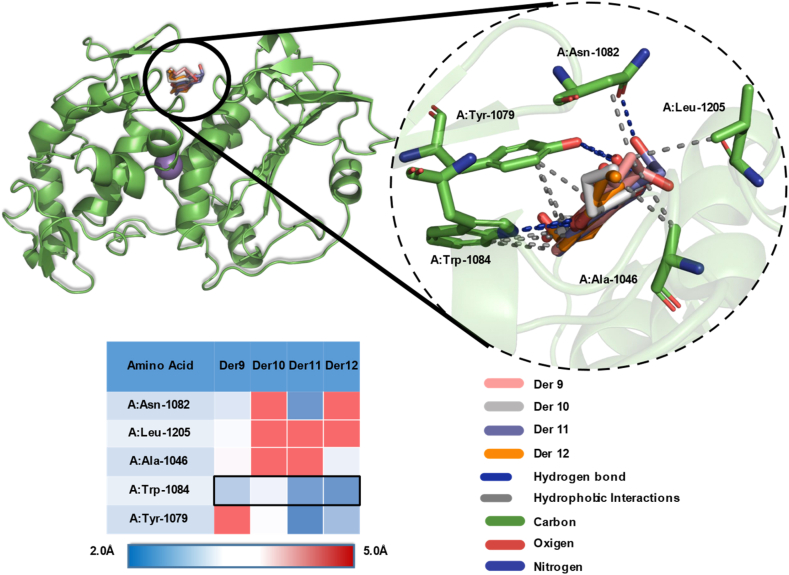
3D interaction modes of Der9 to Der12 with nsP2.

A further evaluation of the interaction modes between the best ligands (Der9 to Der12) and residues in the target, Fig. 3 and Table 1, shows the influence of the stereoisomerism and alkyl group in the carbon 2. Der9 (R and methyl group) has the lowest energy affinity although it has 5 interactions, which they are predominantly hydrophobic and weak (distance >3 ​Å). Der10 (R and propyl group) has 4 interactions, one of which is moderately strong (A:Trp1084, distance ​= ​2.14 ​Å), responsible for it binding. Der11 (S and methyl group) has 6 interactions, 2 weaks (hydrophobic), 2 moderates (hydrogen bond) and 2 strong bonds (hydrogen bonds, A:Tyr-1079, distances: 1.85 ​Å; 1.96 ​Å). Der12 (S and propyl group) has 3 weak hydrophobic interactions and 2 moderate hydrogen bond interactions, reason that has slightly higher binding energy with the ligand than Der9.

**Table 1:** Interactions between the best ligands (Der9 to Der12) and the nsP2 enzyme residues.

Ligand	RMSD (Å)	Receptor	Interaction	Distance (Å)
Der9	1.821	A:Trp-1084	Hydrofobic	3.43
		A:Leu-1205	Hydrofobic	3.78
		A:Asn-1082	Hydrofobic	3.44
		A:Ala-1046	Hydrofobic	3.89
		A:Trp-1084	H-Bond	3.78
Der10	1.865	A:Tyr-1079	Hydrofobic	3.83
		A:Trp-1084	Hydrofobic	3.65
		A:Tyr-1079	H-Bond	3.39
		A:Trp-1084	H-Bond	2.14
Der11	1.416	A:Tyr-1079	Hydrofobic	3.87
		A:Trp-1084	Hydrofobic	3.86
		A:Tyr-1079	H-Bond	1.85
		A:Tyr-1079	H-Bond	1.96
		A:Asn-1082	H-Bond	2.08
		A:Trp-1084	H-Bond	2.21
Der12	1.625	A:Ala-1046	Hydrofobic	3.60
		A:Tyr-1079	Hydrofobic	3.99
		A:Trp-1084	Hydrofobic	3.61
		A:Tyr-1079	H-Bond	2.73
		A:Trp-1084	H-Bond	2.06

It's important to note the influence of the stereoisomerism and alkyl group in the residues interactions, as shown in Table 1, an S isomery and methyl group has greater tendency to output the most interactions.

**Fig. 4:**
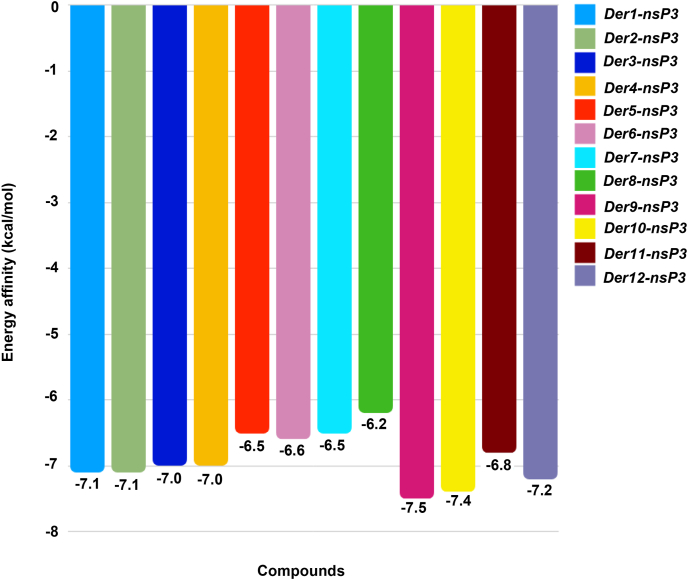
Energy affinity for the complex nsP3-ligands (Der1 to Der12).

In Fig. 4, the energy affinity of the ligands and nsP3 is shown. The same criteria for selection were used for selecting the best ligands (energy affinity close to or lower than −6.0 ​kcal/mol). All the ligands have lower energy affinity with nsP3 than nsP2, as expected when comparing with the compounds (pyrimidone derivatives) tested by Zhang [22] through docking simulation, which were able to inhibit the nsP3 and they have some similarities with the chromones and phenol derivatives.

**Fig. 5:**
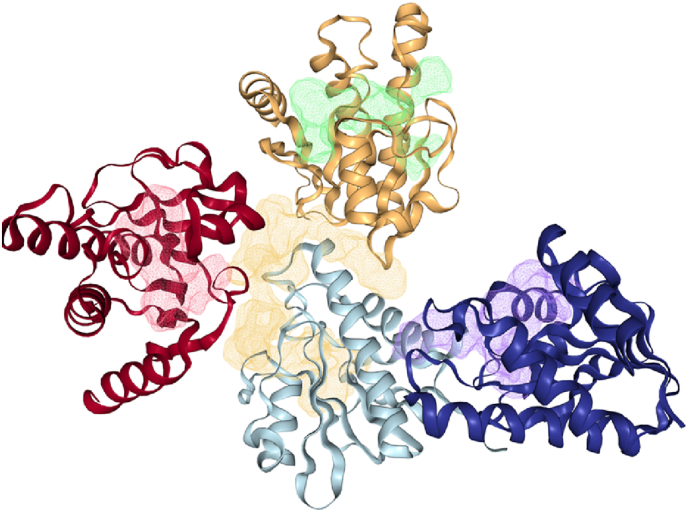
3D binding sites of the nsP3 enzyme chain A, B, C and D, represented in red, orange, light cyan and blue, respectively.

Fig. 5 represents the possible binding sites of the enzyme 3GPG, DoGSiteScorer is a method based in grid that uses a Gaussian Difference (Difference of Gaussian - DoG) Filter for the detection of possible binding subpockets that is able to fit a sphere-like object, based on the density, a cluster of subpockets forms a pocket. Subsequently, physico-chemical descriptors are calculated to determine the volume, surface area, lipophilicity, that are used to calculate the simple score of each pocket [46].

In Fig. 5 its clearly stand out the central pocket (yellow), that pervades all the chains, with a simple score of 0.63 due to the high volume (1924.48 ​Å^3^) and surface area (2328.39 ​Å^2^). The second highest score, 0.61, is in the D chain (blue), the lilac pocket, with 949.4 ​Å^3^ of volume and 1044.95 ​Å^2^ of surface area. The third (green), 0.57, is in the B chain (orange), with 866.67 ​Å^3^ and 1077.46 ​Å^2^ and the last (red), 0.45, in the A chain (red) has 653.74 ​Å^3^ and 748.74 ​Å^2^. Although there's a higher probability of interaction the yellow pocket, the size of the ligands must be accounted for, so in the case of a small ligand that can fit in a small pocket may have a suitable interaction with its surrounding, while a large pocket size relative to a small ligand may indicate a greater distance of interaction, in contrast to the nsP2 simulation.

**Fig. 6:**
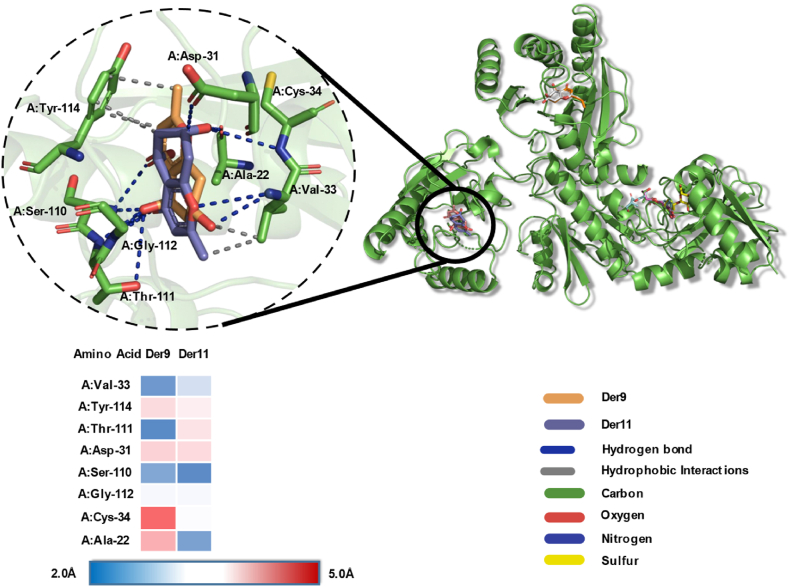
3D interaction of the complex enzyme nsP3-ligands Der9 and Der11, represented in orange and lilac respectively.

The interaction between the ligands Der9 and Der11, the two best ligands (−7.5 ​kcal/mol and −7.4 ​kcal/mol), and the nsP3 residues are presented in Fig. 6 (which highlights only the two best for a clearer visualization) and Table 2, which contains only the residues with the best interaction (Der9 and Der10) and their analogues (Der11 and Der12), included due to the similarity and for comparison to the nsP2 enzyme. The main residues for the replication process of CHIKV are Asp 10, Ile 11, Asn 24, Asp 31, Gly 32, Val 33, Cys 34, Ser 11, Tyr 114, Val 133 and Arg 144, as described in the work of Puranik et al. [19]. The Der9 and Der11 docking results corroborate with the residues interaction, which 5 and 3 critical interactions, respectively.

**Table 2:** Interactions between the best ligands and their analogues (Der9 to Der12) with nsP3 enzyme residues.

Ligand	RMSD (Å)	Amino Acid	Interaction	Distance (Å)
Der9	1.456	**A:Val-33**	**Hydrofobic**	**3.77**
		**A:Tyr-114**	**Hydrofobic**	**3.39**
		**A:Asp-31**	**H-Bond**	**3.52**
		**A:Val-33**	**H-Bond**	**2.28**
		**A:Ser-110**	**H-Bond**	**2.37**
		A:Thr-111	H-Bond	2.19
		A:Gly-112	H-Bond	1.86
Der10	1.650	B:Ala-22	Hydrofobic	3.50
		**B:Asn-24**	**Hydrofobic**	**3.82**
		**B:Val-33**	**Hydrofobic**	**3.73**
		B:Val-113	Hydrofobic	3.59
		**B:Tyr-114**	**Hydrofobic**	**3.47**
		**B:Ser-110**	**H-Bond**	**2.98**
		B:Thr-111	H-Bond	1.86
		B:Gly-112	H-Bond	2.26
Der11	1.193	A:Val-33	Hydrofobic	3.49
		**A:Tyr-114**	**Hydrofobic**	**3.72**
		A:Ala-22	H-Bond	2.34
		**A:Val-33**	**H-Bond**	**2.74**
		A:Cys-34	H-Bond	2.94
		**A:Ser-110**	**H-Bond**	**2.20**
		A:Thr-111	H-Bond	2.37
		A:Gly-112	H-Bond	2.89
Der12	1.574	**B:Asn-24**	**Hydrofobic**	**3.81**
		**B:Val-33**	**Hydrofobic**	**3.68**
		B:Val-113	Hydrofobic	3.51
		**B:Tyr-114**	**Hydrofobic**	**3.62**
		**B:Ser-110**	**H-Bond**	**1.93**
		B:Thr-111	H-Bond	1.82
		B:Gly-112	H-Bond	2.32
		**B:Tyr-114**	**Pi Stacking T**	**4.91**

Note: in bold, the residues that play an important role in the CHIKV replication cycle [19].

As mentioned before, the main protein chains of nsP3 involved in interactions are the smallest, and their interactions have high potential of inhibition, it's an important contrast when compared to nsP2, while the ligands can fit in the yellow and lilac pockets, the distances are relatively long, decreasing the binding affinity. Also, a big pocket to a small ligand may increase instability of the binding in time, due to weak interactions with target, possible interactions with surroundings and freedom of movement, as in a small pocket site the opposite can be true, the ligand has higher probability to be entrapped because of the strong interactions and less degrees of freedom.

In comparison, in the work of Seyedi et al. [46] the complex of nsP3 with ADP-ribose (PDB: 3GPO) has a binding affinity is −8.7 ​kcal/mol, obtained through AutoDock Vina and equal to the original XRD structure, which is attributed to a larger molecule and HB receptors, able to interact with most of the same residues as the derivatives presented in Table 2 (Ala22, Asn24, Asp31, Val33, Cys34, Thr111, Gly112, Val113, Tyr114). The disparity in binding affinity may be due to the degree of freedom, the electron affinity and number of HB sites.

Although Der8 has the lowest affinity for the target, −6.2 ​kcal/mol, it's interesting to note that, in the original work of the compounds, presented *in vitro* inhibitory action against ZIKV and still is inside the −6.0 ​kcal/mol against CHIKV, making it a candidate for an *in silico* simulation targeting ZIKV proteins or further studies *in vitro* to discover or propose a mechanism of inhibition and the *in vitro* test of Der8 and CHIKV to develop as a potential drug to treat zika and chikungunya, as they share the vector and some symptoms. For an even further study, both simulations and *in vitro* assays should be made for dengue.

### 3.2 ADMET

As described by Wager and coworkers [47], more polar (40 ​< ​TPSA ≤90 ​Å^2^), less lipophilic (logP ≤3), less basic and larger than commercially available drugs from data sets, including from Pfizer [48], are less likely to be toxic in the nervous system *in vivo*. From those sets, some of the drugs are propofol (a phenol derivative), caffeine, clonazepam, modafinil, alprazolam, quetiapine, fluoxetin, escitalopram and zolpidem, which are all permitted in Brazil under prescription or used as general anesthetics [49], with the exception of caffeine, from coffee drinks and anti-inflammatory drugs.

**Fig. 7:**
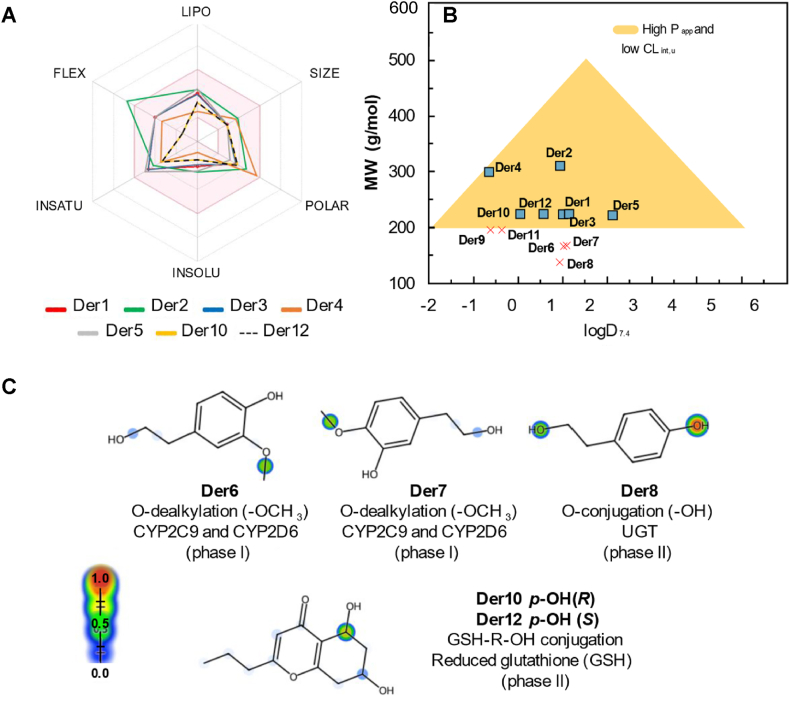
(**a**) Oral bioavailability radar with physicochemical properties of the most favorable ligands in relation to the druglikeness criteria: LIPO (logP <5), SIZE (200 < MW ​< ​500 ​g/mol), POLAR (20 ​< ​TPSA <120 ​Å^2^), INSOLU (logD <4), INSATU (Fsp3 ​> ​0.5) and FLEX (RB ​< ​10). (**b**) Alignment between MW and logD to estimate the ideal physicochemical space for P_app_ and CL_int,u_ descriptors. (**c**) Site of metabolism prediction for the Der6 to Der8, Der10 and Der12 compounds.

In the oral bioavailability radar in Fig. 7a, it's possible to observe the physicochemical properties of the compounds Der1 to Der12 applied to the druglikeness criteria of Pfizer and GSK. The properties clearly show compounds of polarity within the ideal limit predicted by the 2016 Pfizer rule [39] due to low logP and high TPSA values, due to the solvent extraction of the fermented material of *Daldinia* sp. with ethyl acetate, as should be expected. The high hydroxylated, ether, phenolic and chromone groups also contribute to a high number of hydrogen bonding sites (HBA and HBD), relatively to the size and MW of those molecules.

The druglikeness in Table 3, most of the molecules presents high QED values (above 0.6, in a range of 0.0–1.0), the main cause are the low molecular size (MW ​< ​300.12 ​g/mol) and low lipophilicity (logP <2), with the exception of Der4, owing this to the most different molecular structure (highest TPSA, lowest number of logP and second highest MW), indicating that the Der1 to Der3 and Der5 to Der12 are similar to already known and well characterized drugs from the databases used by ADMET consensual prediction. In the MCE-18, it's possible to observe that scores ≥40 suggest that the compounds Der4, Der9, Der10, Der11 and Der12 present an Fsp3 of at least 50% (Fsp3 ​≥ ​0.5), which basically follows the trends observed in medicinal chemistry, where it's worth highlighting the compounds Der10 and Der12 as leading compounds in occupying the ideal physicochemical space (Fig. 7a) [50].

**Table 3:** Physicochemical Properties of Der1 to Der12, in which were applied the GSK, Pfizer and Golden Triangle criteria for druglikeness.

Prop.	Derivatives											
	1	2	3	4	5	6	7	8	9	10	11	12
*Physicochemical properties*												
logP	1.12	1.12	1.31	−0.61	1.83	0.61	0.69	0.77	−0.28	0.55	−0.37	0.50
logD	1.01	0.94	1.13	−0.65	2.13	1.02	1.09	0.94	−0.62	0.05	−0.36	0.57
MW	224.1	310.1	224.1	300.1	222.1	**168.0**	**168.0**	**138.0**	**196.0**	224.1	**196.0**	224.1
TPSA	66.76	93.06	66.76	119.6	49.69	49.69	49.69	40.46	70.67	70.67	70.67	70.67
*Druglikeness criteria*												
Pfizer rule∗	(−)	(−)	(−)	(−)	(−)	(−)	(−)	(−)	(−)	(−)	(−)	(−)
GSK filter	(−)	(−)	(−)	(−)	(−)	(−)	(−)	(−)	(−)	(−)	(−)	(−)
GT rule	(−)	(−)	(−)	(−)	(−)	(+)	(+)	(+)	(+)	(−)	(+)	(−)
QED	0.73	0.70	0.73	0.46	0.71	0.70	0.70	0.63	0.62	0.78	0.62	0.78
F*sp*^3^	0.41	0.50	0.41	0.57	0.38	0.33	0.33	0.25	0.50	0.58	0.50	0.58
MCE-18	14.0	18.0	14.0	44.90	14.0	6.0	6.0	5.0	42.4	41.05	42.4	41.05

**Note:** The prediction was made using the ADMETlab 2.0 platform, where the + tokens indicate an unfavorable druglikeness attribute; in bold, the descriptor that has at least one alert from the rules. ∗Pfizer's rule relates logP and TPSA attributes to the physical-chemical space of the ligands: low logP and high TPSA (logP <3 and TPSA >75 ​Å^2^), low toxic risk; high logP and low TPSA (logP >3 and TPSA <75 ​Å^2^), toxic risk. In blue: desirable values, in red: undesirable. Some descriptors are described in the Supplementary information.

Table 4 brings the pharmacokinectics descriptors of the derivatives, which the results expressed in terms of percentage are considered excellent or moderate when between 30% and 70% (-token) and poor above 70% (+token). A high Madin-Darby Canine Kidney cells passive permeability (P_app_ MDCK) is related to high oral bioavailability, with standard threshold value of 2 × 10^−6^ ​cm ​s^−1^, below that would be estimated to have a poor oral absorption. The results are as expected because of the size and logD_7.4_, as described in the work of Johnson and coworkers [43]. It's worth highlighting that the compounds with the best druglikeness scores, that is, Der10 and Der12, also obtained the best fit to the golden triangle rule (GT rule), especially due to the lowest logD values of positive order (0.05 and 0.57, respectively), within a range of MW 200–500 ​g/mol (Fig. 7b), with P_app_ MDCK values on the order of 1.6 × 10^−5^ and 2.3 × 10^−5^ ​cm ​s^−1^, respectively.

**Table 4:** Predicted ADMET properties for Der1 to Der12 compounds.

Prop.	Derivatives											
	1	2	3	4	5	6	7	8	9	10	11	12
*Absorption*												
P_app_∗ (× 10^−5^)	2.1	2.5	2.1	3.7	1.4	1.5	1.5	1.6	11	1.6	13	2.3
P-gp (%)	(−) 5.4	(−) 0.1	(−) 2.7	(−) 4.5	(−) 0.6	(−) 3.9	(−) 3.9	(−) 0.3	(+) 99.7	(+) 99.9	(+) 99.7	(+) 99.8
HIA (%)	73.0	71.3	73.2	72.0	73.1	73.2	74.5	73.6	73.2	72.9	73.2	72.9
*Distribution*												
VD∗	0.77	0.82	0.67	0.74	1.86	1.24	1.27	3.17	1.01	1.02	0.94	0.81
PPB (%)	56.6	55.3	58.3	38.9	49.1	42.8	42.5	38.9	44.2	46.8	44.2	46.8
BBB (%)	24.4	27.1	24.8	22.2	31.3	33.3	33.2	28.9	30.2	33.0	30.2	33.0
*CYP450 metabolism*												
2C9	(+)	(−)	(+)	(−)	(−)	(+)	(+)	(+)	(+)	(+)	(−)	(+)
2D6	(−)	(−)	(−)	(−)	(+)	(+)	(+)	(−)	(−)	(−)	(−)	(−)
3A4	(−)	(−)	(−)	(−)	(−)	(−)	(−)	(−)	(−)	(−)	(−)	(−)
*Excretion*												
CL_int,u_∗	15.1	12.2	14.8	2.5	12.2	12.6	12.8	13.4	3.3	3.4	4.7	4.7
*Organic Toxicity*												
HT (%)	(−) 13.1	(−) 18.6	(−) 7.2	(−) 04.5	(−)	(−) 9.4	(−)	(−) 4.8	(+) 84.4	(+) 95.3	(+) 73.3	(+) 91.9
					27		12					
Ames (%)	(−)	(−)	(−) 12.2	(−) 30.2	(−) 12.5	(−) 17.7	(−) 15.9	(−) 10.3	(−) 18.3	(−) 33.2	(−)	(−) 19.4
	9.4	3.4									5.9	

**Note:** The prediction was made in a consensual test between the ADMETlab 2.0 and ADMETboost platforms, where the + tokens indicate probability >0.7 of the ADMET attribute being unfavorable. ∗P_app_ are expressed in × 10^−5^ ​cm/s in MDCK cell model, VD are expressed in L/kg and CL_int,u_ is expressed in mL/min/kg. In blue: desirable values, in red: undesirable, the metabolism (values in black) is the exception as it may produce toxic metabolites.

As empirical data and literature reports, low molecular weight compounds usually are easily metabolized, nonetheless, some reactive metabolites can be originated in second phase metabolism, especially in aromatic structures [51]. With the GT rule plot, it was possible to observe that compounds Der6 to Der8 are more displaced from the ideal physicochemical space for good metabolic stability, due to MW ​< ​200 ​g/mol (Fig. 7b), being compounds more susceptible to metabolism of phase I (by CYP450) and phase II (by UGT). With the prediction of the site of metabolism, it was possible to observe that the compounds Der6 and Der7 have MW ​< ​200 ​g/mol and are O-dealkylated by the CYP450 isoforms 2C9 and 2D6 due to the presence of a highly specific -OCH3 group, while the absence of this functional group increases the susceptibility of Der8 to undergo O-conjugation (-OH groups) by the UGT in phase II of metabolism. Despite the presence of metabolism sites, the compounds do not pose a risk of human hepatotoxicity (HT) or Ames mutagenicity due to the formation of reactive secondary metabolites (Table 4).

On the other hand, it's important to highlight that the compounds Der10 and Der12, despite the stereoisomeric difference in the p-OH position, can undergo conjugation with reduced glutathione (GSH) of the GSH-R-OH type in their o-OH groups, constituting a metabolite capable of covalently interacting with proteins and DNA (Fig. 7c), presenting a structural warning regarding the HT model (Table 4).

The low probability of being a P-glycoprotein (P-gp) substrate of Der1 to Der8 indicates that these molecules can be easily absorbed in the intestine, in conform with elevated human intestine absorption (HIA), with HIA values estimated to be at least 70% (Table 4). In contrast to Der9 to Der12, which the P-gp may favors the excretion. It should clearly reflect in the clearance rate and half-life, although if safe for use as an efficient drug to treat chikungunya, novel methods of drug delivery can be used to bypass this, as example, in the form of a delayed release drugs, functionalization or core and shell nanoparticles with biocompatible molecules or materials [52].

The plasma protein binding (PPB) indicates the interaction of the molecules with the proteins in blood serum, therefore an important descriptor for distribution and excretion, related to oral bioavailability. Due to the lipophilic character of these proteins and high polarity of the derivatives, the PPB is low (<90%), showing that they can be transported with ease along the body. It's related with the volume distribution (VD), that corroborates with the PPB. Also, the capability to cross the blood-brain barrier (BBB) to act in the CNS, is low, due to the low lipophilicity [48], similar to PPB. Should be noted that ADMETlab 2.0 express BBB in terms of cm/s, were as the logBB ​> ​−1 is classified as BBB+ and logBB ​≤ ​−1 is classified as BBB-, the result expressed in Table 4 is the probability of BBB+.

The ADMET properties found for Der8 are similar to those in literature, with rapid oral absorption and excretion in 8h in rats [53], phase II UGT and ortho-sulphate (SULT) metabolic pathways, in which the sulphated product of tyrosol (hydroxytyrosol acetate sulphate) [54] may protect cells from oxidized cholesterol and enhances the functionalities of HDL cholesterol due to antioxidant property. Nonetheless, in the case of Chikungunya, a higher care must be made, as a false diagnostic for hemorrhagic dengue may lead to worsening of the clinical condition of the patient as it reduces the viscosity of the blood [55].

It should be noted that tyrosol and their natural derivatives have confirmed and well reported with health benefits, consumed or extracted mostly from extra virgin olive oil, with no significant finding on toxic effects *in vivo* [[56], [57]].

## 4 Limitations and future perspectives

Future *in silico* and *in vitro* evaluations and assays must be made to assert the properties of the derivatives, as should be noted that the ADMET results does not necessarily reflect the actual behavior of the compounds, which is the reason that is suggested to test the derivatives (with the exception of Der8, which is already extensively well studied) to compare with the ADMET results. It is suggested for future works the docking validation [46], molecular dynamics simulation, molecular mechanic calculations [58,59] and density functional theory calculations [60,61].

The results presented in this work are the main findings of the derivatives, due to selection criteria and best candidates, not all results of molecular docking are presented.

## 5 Conclusion

The natural phenolic and chromone derivatives presented mild to low potential to inhibit the nsP2, but outstanding potential to inhibit the nsP3 enzyme of CHIKV. While the ADMET results shows that the best docking molecules may have some drawbacks, the Der8 is highlighted as one of the best candidates, with high QED with good absorption, distribution, excretion, metabolism and toxicity well reported and safe to use. But one of its advantages over the others are that it’s already effective *in vitro* against ZIKV, a disease that shares the same vector and some symptoms. As Der8 shows potential as a natural compound for treating chikungunya and zika, further progression to *in vitro* and *in vivo* experiments is warranted. Additionally, *in silico* and experimental investigations against the dengue virus could be valuable for addressing these co-circulating endemic diseases.

## CRediT authorship contribution statement

**Joan Petrus Oliveira Lima:** Methodology, Formal analysis, Data curation. **Caio Henrique Alexandre Roberto:** Software, Methodology, Investigation. **Matheus Nunes da Rocha:** Software, Methodology, Investigation, Formal analysis. **Victor Moreira de Oliveira:** Investigation, Formal analysis, Data curation, Conceptualization. **Rafael Melo Freire:** Visualization, Resources. **Ralph Santos-Oliveira:** Visualization, Resources, Funding acquisition. **Emmanuel Silva Marinho:** Writing – review & editing, Writing – original draft, Validation, Supervision, Conceptualization. **Pedro de Lima Neto:** Visualization, Validation, Supervision. **Pierre Basílio Almeida Fechine:** Writing – review & editing, Visualization, Supervision, Project administration.

## Ethics approval

Not applicable.

## Declaration of generative AI

The Authors declare that no generative AI have been used throughout the entire writing process of this manuscript.

## Funding information

This work was supported by 10.13039/501100003593CNPq (308452/2022-4), 10.13039/501100002322CAPES (Finance Code 001- PROEX 23038.000509/2020–82) and Fondecyt 11200425, 124117 and AFB220001.

## Data availability

The following softwares and webservers were used: PubChem (https://pubchem.ncbi.nlm.nih.gov/), PDB nsP2 (https://www.rcsb.org/structure/3TRK), PDB nsP3 (https://www.rcsb.org/structure/3GPG), AutoDock Vina (https://vina.scripps.edu/), DoGSiteScorer (https://proteins.plus/), AutoDock Tools (https://autodocksuite.scripps.edu/adt/), MarvinSketch (https://chemaxon.com/marvin), ADMETlab 2.0 (https://admetmesh.scbdd.com/), ADMETboost (https://ai-druglab.smu.edu/admet), SwissADME (http://www.swissadme.ch/), AI Drug Lab (https://ai-druglab.smu.edu/), XenoSite (https://xenosite.org/), pkCSM (https://biosig.lab.uq.edu.au/pkcsm/), ProTox II (https://tox-new.charite.de/protox_II/).

## Declaration of competing interest

The authors declare that they have no known competing financial interests or personal relationships that could have appeared to influence the work reported in this paper.
