# Supplementary material for: Phenol and chromone compounds for in silico inhibition of nsP2 and nsP3 of Chikungunya virus
Source: Pharm Sci Adv. 2025 Jul 25;3:100084. doi: 10.1016/j.pscia.2025.100084 (PMC12709867; doi:10.1016/j.pscia.2025.100084)
Supplement: Multimedia component 1 [file mmc1.docx]

**SUPPLEMENTARY MATERIAL**

**Phenol and chromone compounds for *in silico* inhibition of nsP2 and nsP3 of Chikungunya virus**

Joan Petrus Oliveira Lima^a^, Caio Henrique Alexandre Roberto^b^, Matheus Nunes da Rocha^b^, Victor Moreira de Oliveira^b^, Rafael Melo Freire^c^, Ralph Santos-Oliveira^d^, Emmanuel Silva Marinho^a,b^, Pedro de Lima Neto^a^ and Pierre Basílio Almeida Fechine^a,*^

*^a^Advanced Materials Chemistry Group (GQMat), Department of Analytical Chemistry and Physical Chemistry, Federal University of Ceará – UFC, Campus do Pici, CP 12100, ZIP Code 60451-970, Fortaleza, CE, Brazil*;

*^b^* *Programa de Pós-Graduação em Ciências Naturais, Universidade Estadual do Ceará – UECE, Centro de Ciências e Tecnologias – CCT, Av. Dr. Silas Munguba, 1700 – Campus do Itaperi, Fortaleza, CE, CEP 60.714-903, Brazil;*

*^c^ Universidad Central de Chile, 8330601 Santiago, Chile;*

*^d^ Brazilian Nuclear Energy Commission, Nuclear Engineering Institute, Laboratory of Nanoradiopharmaceuticals and Synthesis of Novel Radiopharmaceuticals, Rio de Janeiro 21941906, Brazil;*

Corresponding author: Pierre Basílio Almeida Fechine

*e-mail: [fechine@ufc.br](mailto:fechine@ufc.br)

**ADMET**

**Table 1.** Physicochemical Properties of Der1 to Der12, in which were applied the GSK, Pfizer and Golden Triangle criteria for druglikeness.

| **Prop.** | **Derivatives** | | | | | | | | | | | |
| --- | --- | --- | --- | --- | --- | --- | --- | --- | --- | --- | --- | --- |
|  | **1** | **2** | **3** | **4** | **5** | **6** | **7** | **8** | **9** | **10** | **11** | **12** |
| *Physicochemical properties* | | | | | | | | | | | | |
| logP | 1.12 | 1.12 | 1.31 | -0.61 | 1.83 | 0.61 | 0.69 | 0.77 | -0.28 | 0.55 | -0.37 | 0.50 |
| logD | 1.01 | 0.94 | 1.13 | -0.65 | 2.13 | 1.02 | 1.09 | 0.94 | -0.62 | 0.05 | -0.36 | 0.57 |
| MW | 224.1 | 310.1 | 224.1 | 300.1 | 222.1 | **168.0** | **168.0** | **138.0** | **196.0** | 224.1 | **196.0** | 224.1 |
| HBA | 4 | 6 | 4 | 7 | 3 | 3 | 3 | 2 | 4 | 4 | 4 | 4 |
| HBD | 2 | 2 | 2 | 5 | 2 | 2 | 2 | 2 | 2 | 2 | 2 | 2 |
| TPSA | 66.76 | 93.06 | 66.76 | 119.6 | 49.69 | 49.69 | 49.69 | 40.46 | 70.67 | 70.67 | 70.67 | 70.67 |
| RB | 6 | 10 | 6 | 5 | 6 | 3 | 3 | 2 | 0 | 2 | 0 | 2 |
| NR | 1 | 1 | 1 | 2 | 1 | 1 | 1 | 1 | 2 | 2 | 2 | 2 |
| SC | 1 | 2 | 1 | 5 | 1 | 0 | 0 | 0 | 2 | 2 | 2 | 2 |
| *Druglikeness criteria* | | | | | | | | | | | | |
| Pfizer rule* | (–) | (–) | (–) | (–) | (–) | (–) | (–) | (–) | (–) | (–) | (–) | (–) |
| GSK filter | (–) | (–) | (–) | (–) | (–) | (–) | (–) | (–) | (–) | (–) | (–) | (–) |
| GT rule | (–) | (–) | (–) | (–) | (–) | (+) | (+) | (+) | (+) | (–) | (+) | (–) |
| QED | 0.73 | 0.70 | 0.73 | 0.46 | 0.71 | 0.70 | 0.70 | 0.63 | 0.62 | 0.78 | 0.62 | 0.78 |
| F*sp*^3^ | 0.41 | 0.50 | 0.41 | 0.57 | 0.38 | 0.33 | 0.33 | 0.25 | 0.50 | 0.58 | 0.50 | 0.58 |
| MCE-18 | 14.0 | 18.0 | 14.0 | 44.90 | 14.0 | 6.0 | 6.0 | 5.0 | 42.4 | 41.05 | 42.4 | 41.05 |

**Note:** The prediction was made using the ADMETlab 2.0 platform, where the + tokens indicate an unfavorable druglikeness attribute; in bold, the descriptor that has at least one alert from the rules. *Pfizer's rule relates logP and TPSA attributes to the physical-chemical space of the ligands: low logP and high TPSA (logP < 3 and TPSA > 75 Å²), low toxic risk; high logP and low TPSA (logP > 3 and TPSA < 75 Å²), toxic risk.
